# The critical view of safety during laparoscopic cholecystectomy: Strasberg Yes or No? An Italian Multicentre study

**DOI:** 10.1007/s00464-020-07852-6

**Published:** 2020-08-11

**Authors:** Lucia Ilaria Sgaramella, Angela Gurrado, Alessandro Pasculli, Nicola de Angelis, Riccardo Memeo, Francesco Paolo Prete, Stefano Berti, Graziano Ceccarelli, Marco Rigamonti, Francesco Giuseppe Aldo Badessi, Nicola Solari, Marco Milone, Fausto Catena, Stefano Scabini, Francesco Vittore, Gennaro Perrone, Carlo de Werra, Ferdinando Cafiero, Mario Testini, SYoN Italian Collaborative Group, Gian Luca Baiocchi, Gianandrea Baldazzi, Mario Battocletti, Sergio Bertoglio, Paolo Bianco, Walter Bugiantella, Giovanni Ciaccio, Lorenzo Cobianchi, Giovanni Conzo, Michele Crespi, Michele De Rosa, Giovanna Di Meo, Ludovico Docimo, Luca Fabris, Cosimo Feleppa, Valentina Ferraro, Tommaso Fontana, Claudio Gambardella, Andrea Gennai, Francesco Guida, Laura Invernizzi, Andrea Massobrio, Fabio Medas, Luigi Monaco, Gianfranco Muntoni, Mario Musella, Denise Palombo, Roberto Perinotti, Davide Pertile, Angela Pezzolla, Gianluca Piccirillo, Roberto Polastri, Roberto Ruggiero, Marco Scatizzi, Carlo Somaglino, Salvatore Tolone, Enrico Traverso, Roberta Tutino, Carlo Valduga, Michele Zuolo

**Affiliations:** 1 Unit of General Surgery “V. Bonomo”, Department of Biomedical Sciences and Human Oncology, University of Bari “Aldo Moro”, Policlinico, Piazza Giulio Cesare, 11, 70124 Bari, Italy; 2 Department of Digestive Surgery, Assistance Publique Hôpitaux de Paris, Henri Mondor Hospital, Université Paris-Est (UEP), Créteil, France; 3 Department of Emergency and Organ Transplantation, University “Aldo Moro” of Bari, Bari, Italy; 4 Department of General Surgery, “Sant’Andrea” Hospital La Spezia, La Spezia, Italy; 5 Division of General Surgery, Department of Surgery, San Donato Hospital, via Pietro Nenni 20-22, 52100 Arezzo, Italy; 6 Department of General Surgery, Cles Hospital, Cles, Italy; 7 Department of General Surgery, “Clinica Sant’Elena” - Quartu Sant’Elena, Quartu Sant’Elena, Italy; 8 Department of Surgery, IRCSS Ospedale Policlinico San Martino, Genova, Italy; 9 Department of Clinical Medicine and Surgery, Federico II” University, Napoli, Italy; 10 Department of Emergency and Trauma Surgery, Parma University Hospital, Parma, Italy

**Keywords:** Cholecystectomy, Critical view of safety, Laparoscopy, Bile duct injuries, Intraoperative bleeding, Laparoscopic training

## Abstract

**Background:**

Laparoscopic cholecystectomy is considered the gold standard for the treatment of gallbladder lithiasis; nevertheless, the incidence of bile duct injuries (BDI) is still high (0.3–0.8%) compared to open cholecystectomy (0.2%). In 1995, *Strasberg* introduced the "Critical View of Safety" (CVS) to reduce the risk of BDI. Despite its widespread use, the scientific evidence supporting this technique to prevent BDI is controversial.

**Methods:**

Between March 2017 and March 2019, the data of patients submitted to laparoscopic cholecystectomy in 30 Italian surgical departments were collected on a national database. A survey was submitted to all members of Italian Digestive Pathology Society to obtain data on the preoperative workup, the surgical and postoperative management of patients and to judge, at the end of the procedure, if the isolation of the elements was performed according to the CVS. In the case of a declared critical view, iconographic documentation was obtained, finally reviewed by an external auditor.

**Results:**

Data from 604 patients were analysed. The study population was divided into two groups according to the evidence (Group A; *n* = 11) or absence (Group B; *N* = 593) of BDI and perioperative bleeding.

The non-use of CVS was found in 54.6% of procedures in the Group A, and 25.8% in the Group B, and evaluating the operator-related variables the execution of CVS was associated with a significantly lower incidence of BDI and intraoperative bleeding.

**Conclusions:**

The CVS confirmed to be the safest technique to recognize the elements of the *Calot* triangle and, if correctly performed, it significantly impacted on preventing intraoperative complications. Additional educational programs on the correct application of CVS in clinical practice would be desirable to avoid extreme conditions that may require additional procedures.

Laparoscopic cholecystectomy (LC) is currently and worldwide considered the gold standard for the treatment of gallbladder lithiasis. Since its introduction, in the early 1990s, this procedure has gained a remarkable consensus until becoming a routine surgical procedure.

LC is characterized by a reduction in postoperative pain, hospital stay, and recovery times to normal daily activities, which translates into reduced costs for the national healthcare systems (NHS) [1]. However, this procedure comes with an increased incidence of bile duct injuries (BDI), compared to open cholecystectomy (OC): 0.3% and 0.8% vs 0.2% [2–7].

LC-related BDIs include minor injuries up to complex hilar injuries, as classified by Strasberg et al., in which the most severe types correspond to type E injuries including ongoing stricture, complete occlusion, resection or division of the bile ducts [8, 9]. The management of BDI may require additional treatments ranging from endoscopic retrograde cholangiopancreatography (ERCP) to restorative surgery, up to hepatic transplantation in selected cases, leading to a significant increase in postoperative morbidity, mortality, and costs. Risk factors of BDI can be divided in patient- and surgery-related [10, 11].

Although the focus in the current literature has been on biliary complications of LC, the risk of intraoperative bleeding has also been reported with a variable incidence in many series and case reports [12, 13].

Intra- or postoperative bleeding in case of LC represents an important, but poorly documented, complication ranging from minor haematomas to significant bleeds (missed operative injuries, slippage of clips) potentially requiring blood transfusion or re-intervention. It has been reported as the most frequent cause of procedure-related mortality in LC (after anaesthesia-related deaths) [13, 14].

The cornerstones for performing a safe cholecystectomy include an adequate knowledge of normal anatomy and related variants, an identification of predictive factors for difficult surgery, and the employment of a correct technique. Since the introduction of laparoscopy, the "infundibular" technique (IT) and the intraoperative recognition of cystic duct and gallbladder junction for gallbladder hilar dissection have been primarily used. In alternative to IT, *Strasberg* introduced in 1995 the "Critical View of Safety" (CVS) to promote the recognition of the gallbladder elements to reduce the risk of BDI and to avoid mistakes due to anatomical alterations and altered visual perception [8]. The importance of the CVS was also recently recognized by the Society of American Gastrointestinal and Endoscopic Surgeons (SAGES), who encouraged the use of this technique in the "Safe Cholecystectomy Program" to minimize BDI risk and promoted the adoption of a universal culture of safety in cholecystectomy (https: // www.sages.org/safe-cholecystectomy-program/). However, despite the widespread use of CVS, a significant BDI decrease has not yet been recorded. Moreover, the scientific evidence supporting this technique to prevent BDI is controversial [15–18]. Several studies, indeed, suggest that the regular use of CVS can reduce or eliminate the risk of BDI; nevertheless, the impossibility to consider a control group burdens the same studies [10, 19]. Meanwhile, other studies contrast the widespread consensus for the technique in the scientific community, showing that CVS is not associated with a useful and correct application in clinical practice [10, 19–23].

This prospective study aimed to assess the impact of the correct application of CVS principles during LC on the incidence of postoperative complications, such as BDI and bleeding.

## Materials and methods

The *SYoN* (Strasberg Yes or No) study is a multicentre Italian observational prospective cohort study, performed by collecting and analysing clinical data of patients managed in 30 Italian surgical departments affiliated with the Italian Digestive Pathology Society (SIPAD), over a study period of 2 years.

All members of SIPAD have been invited by email to participate in the study through an online questionnaire. The questionnaire (23 questions divided into six forms) examined the preoperative workup, the laparoscopic training of the first surgeon, the intraoperative management of the patient, and the postoperative phase concerning any BDI and perioperative bleeding.

All involved centres had a critical volume > 100 laparoscopic cholecystectomies performed per year.

The study was conducted prospectively. The insertion of patients’ data in the national database was performed after patient discharge. Patients, indeed, received the most suitable surgical treatment based on their clinical conditions, the preoperative study and the intraoperative findings.

To ensure standardization among the enrolled centres, these were provided with definitions of pathological obesity (BMI ≥ 30 kg/m^2^), biliary leakage (presence of bile in abdominal drains lesser than 300–500 per day or intra-abdominal collections) [24, 25], bleeding (defined as loss of blood ranging from minor haematomas to significant bleeds that require re-operation or blood transfusions) [13], iatrogenic lesions according to Strasberg classification and CVS.

The CVS was achieved when these three fundamental components were respected: (1) the *Calot* triangle (bordered by the cystic duct, common hepatic duct, and inferior liver edge) is liberated from the surrounding fibrous and fat tissue, (2) the lower third of the gallbladder is separated from the liver up to the visualization of the surface of the liver with evidence of the *Rouviere* sulcus through the dissected area, (3) the sure recognition of two unique structures that enter into gallbladder.

The surgeon was asked to judge personally, at the end of the procedure, if the isolation of the elements was performed according to all the points described by *Strasberg*; subsequently, during questionnaire filling, the surgeon introduced, at the same time, data on pre-, intra- and postoperative patient course attaching an iconographic item (Video or "Doublet Photography") in case of confirmed dissection of the *Calot* triangle with a correct CVS application.

Patients submitted to emergency or elective LC, for acute cholecystitis (AC), chronic pathologies, and during other major laparoscopic surgeries were eligible for inclusion, if a proper preoperative examination was conducted by the operating surgeon. Patients who needed conversion to open surgery or who underwent surgery with evidence of malignant pathologies of the gallbladder were excluded.

**Fig. 1:**
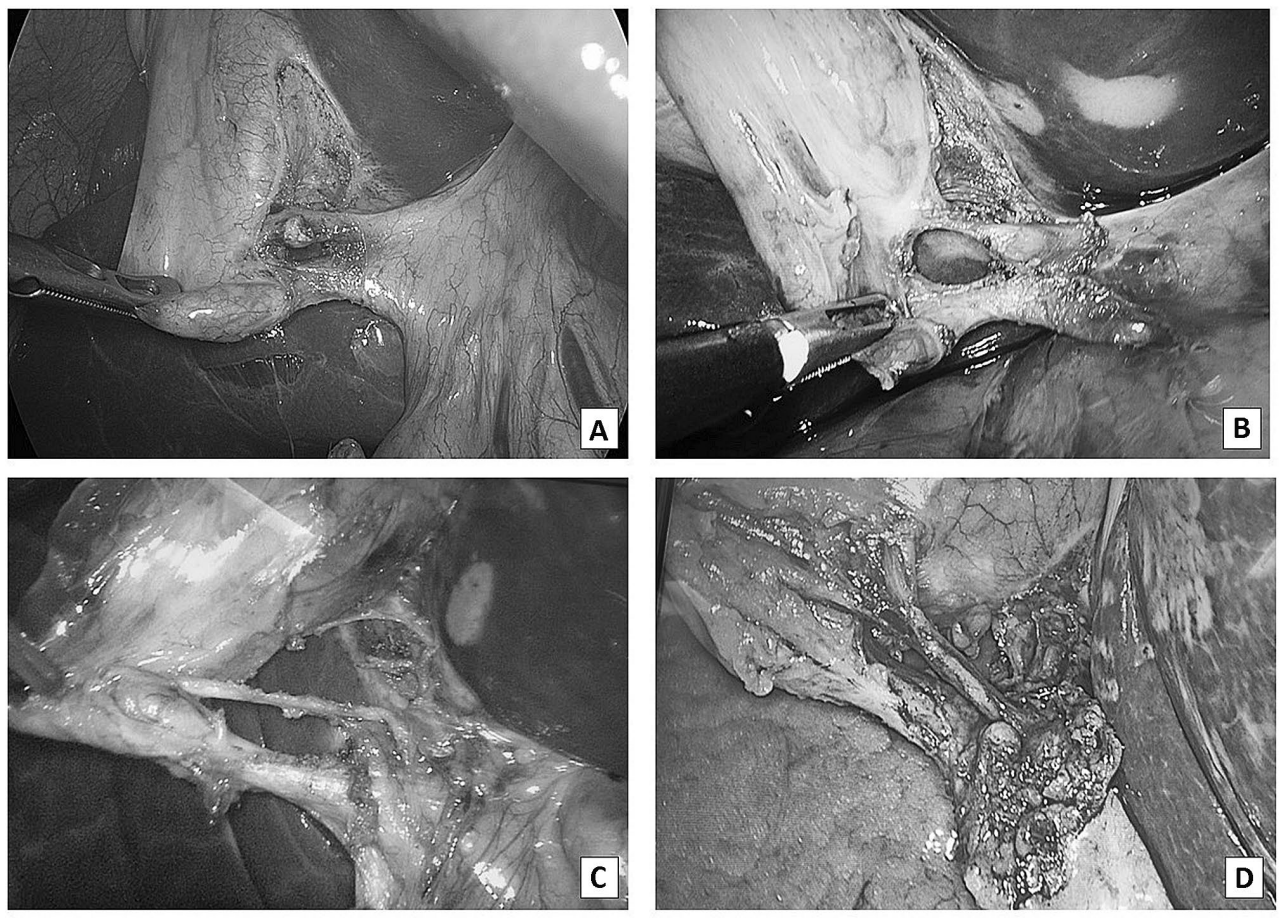
**A**–**D** Intraoperative photo of the Calot’s triangle dissection according to CVS principles and reviewed by the external auditor

During compilation, the iconographic documentation (video or photo) was sent to a dedicated encrypted email address indicating the date of the surgery, the patient's initials, the date of birth and the recruiting centre. Data collection was centrally recorded into an electronic database of the data manager (SIPAD), which also ensured the blinding of the lead operator. Finally, an expert surgeon with high skill in hepatobiliary and laparoscopic surgery reviewed, as external auditor, all the iconographic documentation to establish the strict adherence of the declared manoeuvre with the three principles of the CVS of *Strasberg*. Figure 1 reports some intraoperative photo of the *Calot* triangle dissection according to CVS principles and reviewed by the external auditor.

The Ethics Committee of the University of Bari (Italy) approved the study (protocol n. 5674, 08/06/2018). Since no different interventions were performed, and patients were treated after signing a written consent form for the recording and research use of iconographic documentation, the Ethics Committee exempted it from the Research Involving Human Subjects Act.

The participating centres contributed by enrolling different numbers of patients, also starting the recruitment at different times. The enrolment was stopped once we reached a sufficient sample of patients for statistical analysis. To integrate the data obtained, at the end of the enrolment, all the centres were interrogated on the conversion rate recorded in each unit during the study period.

This study could not be randomized for ethical reasons and was blinded for the operators who analysed the iconographic findings and for the statistician.

### Statistics

The statistical analysis was carried out with STATA14 (StataCorp LLC, College Station, TX, USA). A *p* value < 0.05 was considered statistically significant. The univariate analysis was performed with the *χ*^2^ and Fisher’s exact test, when appropriate. In the analysis, we included covariates known to influence BDI occurrence based on the current literature [17, 26–30]. For instance, we analysed the incidence of sex on BDI based on the experience of *Fullum *et al*.* who reported that men have a higher incidence of BDI after cholecystectomy compared to women [26]; we considered both the abdominal circumference and pathological obesity (BMI > 30 kg/m^2^), as the literature showed that obese patients are 3 times more likely to have a CBD injury as compared to their counterparts [27]. We explored, also, the impact of previous abdominal surgery on BDI based on the historical evidence that prior upper abdominal surgery could be considered as a risk factor for difficult laparoscopic cholecystectomy due to presence of adhesions [28], and the median operative duration as possible expression of surgical difficulty in case of operative durations > 60 min [29]. Finally, according to Tokyo guidelines for acute cholecystitis and WSES guidelines, we discussed the role of acute cholecystitis in influencing BDI incidence [17, 30]. The multivariate analysis was carried out with a logistic regression model, reporting Odds Ratios (OR) and 95% confidence intervals (95% CI) to estimate the effect of the Critical View of Safety on BDI and bleeding by adjusting for the significant variables identified by the univariate analysis.

## Results

Between March 2017 and March 2019, data of 712 patients submitted to LC were collected in the national database. Out of these, 604 were analysed, 330 females (54.6%) and 274 males (45.4%), while 108 (15.2%) were excluded: 9 because of a missed correspondence with the surgeon’s declaration on the CVS employment and the external auditor’s opinion, 64 for uncomplete data and 35 for conversion in open surgery.

### Patient-related risk factors

In the 81.9% (*n* = 495) of patients, the indication for surgery was gallbladder lithiasis; in 18.1% (*n* = 109) surgery was performed for AC. In 8.1% (*n* = 49) surgery was performed within 24 h, and in 91.9% (*n* = 555) patients were managed with deferred urgency. At the time of surgery, 163 patients (27.0%) had notable abdominal adiposity with an abdominal circumference > 88 cm in women and > 102 cm in men, whereas 58 patients reported preoperative pathological obesity (9.6%). Previous upper abdominal surgery was reported in 2.8% patients (*n* = 17), lower abdominal in 29.3% (*n* = 177), and both in only 0.2% (*n* = 1). Among the preoperative parameters evaluated, 8.3% (*n* = 50) of patients had significant comorbidities on admission.

### Surgery-related risk factors

The laparoscopic surgical training obtain relevance in the data investigation and the results highlight that in 4.6% (*n* = 28) of cases surgery was performed by young surgeons with a laparoscopic training of less than 30 LC; in 5.8% (*n* = 35) by surgeons who performed 30 to 50 LC, whereas the great majority of procedures (89.6%) were carried out by experienced surgeons with a training of more than 50 LC. In 51.5% of cases, the duration of surgery exceeded 60 min (range: 25- 240 min). The external auditor reviewed the entire iconographic documentation. The correct application of CVS was observed in 73.7% (*n* = 445) of LC, whereas the non-use of CVS was found in 26.3% (*n* = 159).

### Predictors of complications: BDI and/or bleeding

The study population was then divided into two groups based on the evidence (Group A; *n* = 11, 1.8%) or absence (Group B; *n* = 593, 98.2%) of BDI and perioperative bleeding after LC.

Table 1 summarizes demographic data, preoperative findings, patient-related risk factors, surgery-related risk factors, treatment, and postoperative management of both groups.

**Table 1:** Characteristics of BDI group

BDI (Strasberg classification)	BDI Group (*n* = 5)	CVS	No-CVS
Type A	3	1 lesion conservatively managed by ERCP, and sphincterotomy	2 lesions conservatively managed by ERCP, and sphincterotomy
Type B	/	/	/
Type C	/	/	/
Type D	/	/	/
Type E	2	/	2 lesions located > 2 cm from the upper biliary confluent: End-to-end biliary anastomosis of the common bile duct Hepaticojejunostomy with a trans-anastomotic stent + accidental interruption of right hepatic artery

The non-use of CVS was found in 54.6% of procedures in the Group A, and 25.8% in the Group B.

Considering a subgroup consisting of patients with evidence of BDI alone (BDI group; *n* = 5), one patient (20%) developed BDI in conditions of declared CVS, whereas in the remaining 4 patients (80%), CVS was not applied. Patient who reported a BDI in case of CVS presented a lesion type A managed by ERCP, and sphincterotomy. The subgroup of BDI without the employment of CVS was comprehensive of two complete lesions of the major bile ducts located > 2 cm from the upper biliary confluent (type E), one of which is associated with vascular injury, and two leaks from cystic or accessory ducts (type A) treated with ERCP, and sphincterotomy. Both cases of type E lesions were managed with re-surgery and with an early end-to-end biliary anastomosis of the common bile duct with a trans-anastomotic stent and a hepaticojejunostomy, respectively. This last case reported the association of BDI and vascular injury (accidental interruption of right hepatic artery) supplied by the portal vein and collateral arterial channel pathways (Table 1), as demonstrated by CT scan and liver function blood test.

Among the cohort of 604 patients analysed, 8 (1.3%) cases presented bleeding that was conservatively managed.

No patients with surgical emergency management for AC (109 patients) have developed intra- or postoperative complications. Among these, in majority of cases (67.9%; *n* = 74) a correct CVS application was reported and no patients have been treated by surgeon with less than 30 cholecystectomy performed.

During the enrolment period, all involved centres registered a conversion rate ranging from 3 to 9% (average: 4.9%), and the most common reasons were the need for CBD exploration due to the altered *Calot’s* triangle anatomy, BDI, and/or intraoperative bleeding. Conversion to open surgery were caused by BDI in 5 patients (14.3% of converted cases) and bleeding in one case (2.8%).

No patient died during the study period.

### Univariate and multivariate analysis

By evaluating in univariate analysis (Table 2), the patient-related preoperative variables, the abdominal circumference (> 88 cm in females and > 102 cm in males) emerged, unexpectedly, as a protective prognostic factor for BDI or bleeding (*p* = 0.04). The preoperative diagnosis of AC (*p* = 0.22), the setting of surgery (election or emergency; *p* = 1.0), the history of previous operations (*p* = 0.41) and the pathological obesity (*p* = 0.61) in the analysed sample were not associated with the unfavourable progress of the surgical intervention. On the contrary, the presence of comorbidities (more than 1 comorbidity) appeared to detect a frail sample of population with a worse prognosis and was significantly associated (*p* = 0.05) with intraoperative complications. Concerning the operator-related variables, the laparoscopic training (*p* = 0.70) was not associated with the development of intraoperative complications, whereas the execution of CVS was associated with a significantly lower incidence of BDI and intraoperative bleeding (*p* = 0.03). The multivariate analysis (Table 3) showed that the presence of preoperative comorbidity is a risk factor for BDI and intraoperative bleeding (*p* = 0.003), whereas the employment of CVS played a protective role in preventing intraoperative complications (*p* = 0.04). Together with this, the preoperative comorbidity maintained statistical significance (*p* = 0.003) while the presence of high abdominal circumference lost significance. Finally, considering the univariate analysis on group with perioperative complications, because of the smallness of the sample under examination, the same parameters lost significance.

**Table 2:** Univariate analysis

			Tot (*N* = 604)	Group A: BDI and/or perioperative bleeding (*N* = 11)	Group B: absence of complications (*N* = 593)	P^a^
Patient-related risk factors (%)	Sex	M	274 (45.4)	8 (72.7)	266 (44.9)	0.066
		F	330 (54.6)	3 (27.3)	327 (55.1)	
	Acute cholecystitis		109 (18.1)	0 (0)	109 (18.4)	0.228
	Weight > 75 kg		273 (45.2)	4 (36.4)	269 (45.4)	0.552
	Abdominal circumference (> 88 cm *F*, > 102 cm M)		163 (27.0)	0 (0)	163 (27.5)	**0.042**
	Setting of surgery	Emergency	49 (8.1)	0 (0)	49 (8.3)	1.000
		Election	555 (91.9)	11 (100)	544 (91.7)	
	Previous surgery	Upper abdominal surgery	17 (2.8)	0 (0)	17 (2.9)	0.419
		Lower abdominal surgery	177 (29.3)	1 (9.1)	176 (29.7)	
		Upper and Lower abdominal surgery	1 (0.2)	0 (0)	1 (0.2)	
	Pathological obesity		58 (9.6)	0 (0)	58 (9.8)	0.612
	Comorbidities		50 (8.3)	3 (27.3)	47 (7.9)	**0.050**
Surgeon-related risk factors (%)	Surgeon’s training	< 30	28 (4.6)	0 (0)	28 (4.7)	0.705
		> 30 < 50	35 (5.8)	1 (9.1)	34 (5.7)	
		> 50	541 (89.6)	10 (90.9)	531 (89.5)	
	Duration of surgery > 60 min		311 (51.5)	8 (72.7)	303 (51.1)	0.155
	Strasberg’s CVS	Performed	445 (73.7)	5 (45.4)	440 (74.2)	**0.032**
		Not performed	159 (26.3)	6 (54.6)	153 (25.8)	

Data are given as absolute values and percentages. Group A: BDI and/or perioperative bleeding; Group B: absence of complications

^a^Between-group comparison made using *χ*^2^ and Fisher’s exact test. Bold emphasized values are statistically significant. *P* < 0.05 was considered statistically significant

**Table 3:** Multivariate analysis

	OR (95% CI)	*p*^a^
Abdominal circumference	na	na
Comorbidities	9.02 (2.13–38.28)	0.003
Strasberg’ CVS	0.28 (0.08–0.98)	0.046

OR (95% CI): odds ratio (95%). *p* (< 0.05)

^a^Between-group comparison made using multivariate logistic regression, adjusting ORs for abdominal circumference, comorbidities and Strasberg

## Discussion

Numerous studies have questioned the incidence of BDI during LC by analysing its causes and risk factors and demonstrating how the incidence rate during LC is still double compared to the OC.

In 1992, Morgenstern et al. reported on 1200 consecutive open cholecystectomies a BDI incidence rate < 0.2% and at the same time considered this value the standard on which LC should be compared [31]. In 2003, Flum et al., analysing a North American database of 1,570,361 cholecystectomies, showed that the incidence of BDI was 0.5% and, as confirmed by Way et al., it rose in cases of AC, especially in case of conversions to open surgery with an overall rate of 1.2% [11, 32, 33]. During the first 5 years of LC introduction, the procedure was associated with the occurrence of serious complications, some of which are typical of laparoscopic access and not common to open surgery. At the beginning, indeed, the incidence of duodenal and bowel injuries, due to trocar puncture or coagulation necrosis of the bowel wall resulting in delayed or walled-off perforation, were reported with an incidence rate of 0.07–0.9% (0.04% for duodenal injury). Major vessel and bile duct injury were described with incidence rates up to 4% [8, 11, 34]. Nowadays, this rate is hopefully much lower and ranges between 0.3 and 0.8%, but remains two to three times higher than the injury rates reported for OC [2].

The higher incidence of BDI in LC questioned the appropriate preoperative evaluation of complex cases, the training of surgeons ready to face them and the common risk factors. According to the current literature, the numerous anatomical variants of the biliary tract represent a possible explication of iatrogenic injury but, also pathological obesity, previous surgery on the biliary tract, and an underlying liver disease, may be seen as predisposing factors for perioperative complications [35, 36]. Aziz et al., indeed, on a national database analysis, report that obese patients are three times more likely to have a BDI as compared to their counterparts [27]. Moreover, Kholdebarin et al. [28] report that previous abdominal surgery, especially the upper one, has historically been considered by some authors [37, 38] but not others [39, 40] to be a relative contraindication to laparoscopic cholecystectomy and usually associated with a high risk of BDI due to the presence of adhesions. In this reported study, both pathological obesity and previous abdominal surgery were not associated with an unfavourable surgery. It could be likely interpreted as a random factor, or related to the higher alert required for potentially more technically demanding surgery. Moreover, the significative correlation between different comorbidities and BDI do not find an exhaustive validation in the current literature and should be interpreted as expression of a frail sample of population with a major risk of an adverse surgical outcome [41].

In case of AC, BDI takes place three times more often in patients with severe local conditions due to active AC if compared with patients without inflammation. Indeed, the literature reports that the risk of BDI depends on the severity of the inflammation and the patient's preoperative clinical condition [42–44]. The data analysed in this study, in which no BDI occurred in patients who underwent emergency surgery, are in disagreement with these previously reported data but could be evaluated in consideration of experienced surgeons involvement in the management of potentially difficult cholecystectomies. Contrariwise, these results find validation and confirm in a Cochrane review and other recent observational studies. These studies highlighted, indeed, that early LC (within 48 h) during AC is related to lower surgical complications and lower incidence of BDI, also reducing operative time in comparison to an antibiotic-first approach followed by elective or deferred surgery [45, 46].

In the most recent Tokyo guidelines for AC, the CVS proposed by Strasberg is strongly recommended to prevent BDI. Nevertheless, in case of severe inflammation with subversion of the *Calot* triangle anatomy, the application of CVS could be arduous, leading to consider alternative procedures, such as fundus-first cholecystectomy, subtotal one, or conversion to open surgery [17]. Also, in WSES guidelines for AC, subtotal cholecystectomy and alternative surgical strategy are considered as an important tool in the difficult cholecystectomy [30, 47], useful in case of severe anatomical alteration of *Calot* triangle when surgical dissection and performability of Strasberg manoeuvre is extremely difficult or hazardous (*i.e. Mirizzi* syndrome) [48, 49].

Some scoring tools based on intraoperative findings to identify difficult LC have been suggested, and are increasingly recognized [50]. Indeed, *Sugrue *et al*.* outlines a surgical scoring system incorporating key operative findings to allow grading and standardization of the degree of cholecystitis [51]. Afterwards, Iwashita et al., in the Japan-Korea-Taiwan expert Delphi consensus on surgical difficulty during laparoscopic cholecystectomy, established that the evaluation of the inflammatory tissue surrounding the gallbladder, the state of the *Calot* triangle and the gallbladder bed could offer an objective parameter, and that the use of this scale may be desirable in future studies [52].

When the CVS cannot be safely obtained during dissection of *Calot’s* triangle, conversion to open surgery is advocated to prevent bile duct injury [53]. However, there is a wide variation in the current literature of the conversion rate to open surgery and, in accordance with this reported experience, it ranges from 2 to 15% [54–56]. According to Al Masri et al. [54], surgery-related indications for conversion includes extensive adhesions, significant inflammation, intraoperative difficulty of bile ducts exploration, and obfuscating bleeding. Medical comorbidities (such as pulmonary disease) have been furthermore found to be a risk factor for conversion from laparoscopic to open surgery in different series and for different laparoscopic procedures [57, 58].

Patients undergoing conversion to open surgery show a higher risk of complications and a longer operative time than those who proceeded successfully with LC [59]. Duration of surgery and conversion rate, indeed, has been cited as generic indicators of surgical difficulty, but should also be interpreted as related factors depending on the surgical training and skill of the operator. As suggested by Bharamgoudar et al., the median operative duration of LC is 60 min and some factors were found to be significant independent predictors of long operative durations (> 90 min), including ASA, age, previous surgical admissions, BMI, gallbladder wall thickness and common bile duct diameter [29].

Among the surgeon-related risk factors, the role of laparoscopic training is firmly taken into account in the determination of BDI. Some studies report a higher risk of iatrogenic injury among the first cases of LC performed. Moore et al., in a study on 8,839 cholecystectomies performed by 55 different surgeons with different laparoscopic training, showed that 90% of iatrogenic lesions had occurred within the first 30 cases. Analysis of the data suggests that the risk of injury is 1.7% for the surgeon in the first case of laparoscopic cholecystectomy and drops to 0.17% after the 50th case [60]. This evidence is not verifiable in our case studies because of the small sample of cholecystectomies performed by surgeons with a training < 30 LC. Voitk et al., contrarily to the study mentioned above, suggested that in the learning curve for LC the target to achieve is sufficiently far from the 50 cases, indicating, however, how the surgery time is significantly related with the laparoscopic learning curve and continues to decrease up to 200 cases [61]. Nevertheless a limitation of this study is the absence of correlation between training and iatrogenic lesions. According to other studies, the risk of BDI would not disappear after the first 50 or 200 cases [62]. In a national survey with over 1500 respondents, surgeons reported that about a third of the BDI occurred after 200 cases of LC, demonstrating that injuries could not be related to the surgeon's inexperience but may reflect technical errors [63]. Calvete et al. suggested that no apparent correlation can be found between the surgeon's experience and the incidence of BDI. By analysing 784 patients divided into three groups over a 6-year time period, they showed how the rate of iatrogenic lesions remained similar among the three groups without significant difference [64].

Moreover, LC is primarily based on visual perception, which may be susceptible to errors or misinterpretations. Way et al. analysed 252 BDI, demonstrating that the leading cause of failure, in 97% of cases, was due to the impaired visual perception rather than poor surgical ability [11].

It appears, indeed, that in most cases the iatrogenic lesion is the result of an intentional surgical manoeuvre that results in an unintentional injury, as a section of the biliary tract, and that in the 75% of cases, the injury would not be intraoperatively recognized. Similarly, Dekker and Hugh described how the most common cause of BDI is the erroneous interpretation of visual information during surgery with a failure to recognize the cystic duct misinterpreted as biliary tract [65]. In their series of 49 patients with iatrogenic lesions, 42 patients had injuries caused by incorrect identification of anatomical structures, and in 70% of cases they were not intraoperatively recognized.

The Strasberg’s CVS was introduced with the purpose to overcome errors of interpretation of the visual field during dissection of the elements of the gallbladder, and this is supported by several studies showing that the routinely use of CVS is associated with a reduction or even elimination of BDI [66, 67]. The safety of the manoeuvre described by Strasberg is confirmed by the present study in which it is found as a significant protective factor to prevent BDI and/or haemorrhagic complications. Avgerinos et al. analysed 1,046 patients who underwent LC. No BDI occurred in 998 cases when CVS was performed [1]. However, the study did not include a control group, conversely to our reported research. Although the aim of CVS is to reduce BDI during LC, there was no decrease in countries where its use has now become mandatory. Therefore, it has been hypothesized that CVS is useful in preventing major lesions (Type E) due to complete erroneous recognition of the anatomy, but fails to avoid injuries type A such as biliary fistulas. This is reflected in this study, in which a complete lesion of the major bile ducts located > 2 cm from the upper biliary confluent (type E) is reported among the non-Strasberg group [20, 68].

In recent studies, the growing consensus obtained for CVS in the scientific community has clashed with the evidence that this is not associated with a correct application in clinical practice.

Experienced surgeons with adequate laparoscopic training would often claim to have reached CVS, while intraoperative images would demonstrate the opposite and other studies showed that many respondents, senior surgeons too, were not able to adequately discern the essential steps of this technique [21–23, 68, 69]. This is demonstrated by Nijssen et al., who reported that in disagreement with what was declared by the operators (80% of the surgeons in the analysis stated to carry out the CVS) from the video analysis of the interventions the CVS would be reached only in 10.8% of cases [70]. The number of studies reporting this evidence suggests two possibilities: the iconographic documentation does not correspond to the real intraoperative perception or surgeons who supposed to know CVS in truth fails understanding its application. This would reinforce the concept that programmes and task forces for a safe cholecystectomy help to increase the number of surgeons able to act safely and that the use of additional techniques, such as comparison with iconographic findings, can help the operator to have perception of his work and document what has been done [16, 68, 71–73].

This series appears representative for what concerns the overall complication rate, but not for the analysis of separate outcomes. This limitation could be overcome by enlarging the patient sample, allowing the analysis of the CVS influence on bleeding and BDI separately, and the correlation among AC, CVS employment, and BDI.

## Conclusion

In the present study, some factors that are universally recognized in the current literature as risk factors for bile duct injury, such as acute inflammatory conditions and pathological obesity, were not associated with an increased incidence of iatrogenic lesions if managed with appropriate timing and with the correct surgical approach.

The Critical View of Safety, when correctly applied, is confirmed to be the safest technique for recognizing the elements of the *Calot* triangle, and it is associated with a significant impact in preventing intraoperative complications (iatrogenic lesions and perioperative bleeding). Additional training for the correct application of Critical View of Safety in clinical practice should be desirable to standardize the laparoscopic approach to the gallstone disease.
